# Long-term effects (> 24 months) of multiple lifestyle intervention on major cardiovascular risk factors among high-risk subjects: a meta-analysis

**DOI:** 10.1186/s12872-021-01989-5

**Published:** 2021-04-15

**Authors:** Hilde Bergum, Irene Sandven, Tor Ole Klemsdal

**Affiliations:** 1 Department of Rehabilitation and Lifestyle Medicine, LHL-Hospital Gardermoen, Postboks 103 Jessheimbyen, 2051 Jessheim, Norway; 2 Oslo Centre for Biostatistics and Epidemiology (OCBE), Oslo University Hospital, Oslo, Norway; 3 Department of Preventive Cardiology, Oslo University Hospital, Oslo, Norway

**Keywords:** Lifestyle intervention, Primary prevention, Cardiovascular risk, Hypertension, Hypercholesterolemia

## Abstract

**Background:**

The evidence of the long-term effects of multiple lifestyle intervention on cardiovascular risk is uncertain. We aimed to summarize the evidence from randomized clinical trials examining the efficacy of lifestyle intervention on major cardiovascular risk factors in subjects at high cardiovascular risk.

**Methods:**

Eligible trials investigated the impact of lifestyle intervention versus usual care with minimum 24 months follow-up, reporting more than one major cardiovascular risk factor. A literature search updated April 15, 2020 identified 12 eligible studies. The results from individual trials were combined, using fixed and random effect models, using the standardized mean difference (SMD) to estimate effect sizes. Small-study effect was evaluated, and heterogeneity between studies examined, by subgroup and meta-regression analyses, considering patient- and study-level variables.

**Results:**

Small-study effect was not identified. Lifestyle intervention reduced systolic blood pressure modestly with an estimated SMD of − 0.13, 95% confidence interval (CI): − 0.21 to − 0.04, with moderate heterogeneity (I^2^ = 59%), corresponding to a mean difference of approximately 2 mmHg (MD = − 1.86, 95% CI − 3.14 to − 0.57, *p* = 0.0046). This effect disappeared in the subgroup of trials judged at low risk of bias (SMD = 0.02, 95% CI − 0.08 to 0.11). For the outcome total cholesterol SMD was − 0.06, 95% CI − 0.13 to 0.00, with no heterogeneity (I^2^ = 0%), indicating no effect of the intervention.

**Conclusion:**

Lifestyle intervention resulted in only a modest effect on systolic blood pressure and no effect on total cholesterol after 24 months. Further lifestyle trials should consider the challenge of maintaining larger long-term benefits to ensure impact on cardiovascular outcomes.

**Supplementary Information:**

The online version contains supplementary material available at 10.1186/s12872-021-01989-5.

## Introduction

Despite the decreases in cardiovascular mortality in recent decades, cardiovascular diseases (CVD) are still a leading cause of premature mortality [1, 2]. The risk of CVD strongly relates to modifiable risk factors [3] and a dominant part (50–70%) of the improvement in cardiovascular mortality can be ascribed to risk factor improvements in the population, while approximately 20–40% can be ascribed to better treatments [4, 5].

With convincing evidence relating risk factor levels to CVD morbidity and mortality, most current CVD prevention guidelines include lifestyle intervention as a key element [6–10]. Despite the broad recommendation of lifestyle advice, the long-term effects of multiple lifestyle intervention on cardiovascular risk factors appear sparsely documented.

Systematic reviews and meta-analyses show that lifestyle intervention does result in small reductions in risk factors including blood pressure, cholesterol and smoking when evaluated typically after 3–12 months [11–13]. Nevertheless, studies on lifestyle intervention have not been able to demonstrate a clear impact on coronary heart disease mortality or morbidity [11, 12]. This could be because the risk factor changes observed in studies of short duration are not maintained in the long term [11].

Most lifestyle studies report the effects of lifestyle intervention after 3–18 months, but few studies evaluate the effects beyond this time range. Since improvements in risk factor levels must be maintained over time, i.e. several years, to have an impact on cardiovascular events, it is of interest to elucidate the long-term effects of lifestyle intervention. The aim of this meta-analysis was to assess the long-term effects (i.e. after 24 months) of multiple lifestyle intervention on major cardiovascular risk factors i.e. total cholesterol (TC), systolic blood pressure (SBP) and smoking habits, in subjects with elevated cardiovascular risk from various causes.

## Methods

The study was conducted and reported according to the PRISMA guidelines for meta-analyses and systematic reviews [14]. The protocol was published in the PROSPERO register (https://www.crd.york.ac.uk/prospero/), registration number CRD42018088783.

### Eligibility criteria

We included randomized controlled clinical trials (RCTs) of cardiovascular primary prevention with a follow-up period of at least 24 months. To ensure that the findings would be relevant for the present situation, only studies published after 1990 were considered. Participants included had to be individually randomized.

#### Patients

Eligible studies included patients ≥ 40 years without known CVD, but with at least one criteria of cardiovascular risk: hyperlipidemia, hypertension, cigarette smoking, obesity, inactivity, impaired glucose tolerance, the metabolic syndrome, or diabetes mellitus. Studies which included participants with diabetes mellitus were only included if only a minority (< 50%) of the participants had diabetes and if interventions were directed to reduce cardiovascular risk and not primarily blood glucose levels. Studies were excluded if the inclusion required presence of a specific medical condition.

#### Intervention

The intervention should be a health promotion activity that aimed to reduce total cardiovascular risk; i.e. to reduce more than one cardiovascular risk factor, through behavioral change, primarily related to diet and/or exercise; counseling or educational interventions, and with, or without, stable background pharmacological treatments.

#### Comparison

The studies should have a control group receiving usual care.

#### Outcome variables

Studies had to report the most important cardiovascular risk factors included in major algorithms for assessing total cardiovascular risk; i.e. SBP, TC and, if available, smoking habits.

### Search strategy

A qualified medical librarian at the Medical Library, Oslo University Hospital, was consulted. RCTs published until April 15, 2020 were searched in PubMed, Embase and Cochrane Central Register of Controlled Trials. There were no language or date restrictions. Additional searches were also conducted in Cochrane Database of Systematic Reviews, UpToDate, NICE, and Prospero for ongoing systematic reviews.

In PubMed, Medical Subject Headings (MeSH Major Topic) and words in title were searched alone, or in combination, including lifestyle, primary prevention, combined modality therapy, risk reduction behavior, smoking cessation, diet, exercise, cardiovascular diseases, cardiovascular risk, hypertension, dyslipidemias and hypercholesterolemia. A more restrictive search strategy, including terms expressing lifestyle and cardiovascular risk, was then performed in Embase and Cochrane Central Register of Controlled Trials.

In addition, we manually screened reference lists of eligible papers and relevant systematic reviews.

### Study selection

Two investigators (HB, TOK) independently evaluated studies for possible inclusion. Non-relevant studies were excluded based on title and abstract. The remaining trials were evaluated in full text. Disagreements were resolved by discussion and subsequent consensus.

### Data abstraction

Three reviewers (HB, TOK, IS) independently extracted information from each included trial using a pre-made data extraction form. Country of study origin, number of participants in the intervention and comparison groups, baseline frequency of males and current smokers, mean age of participants, body mass index (BMI), SBP, and TC was extracted.

### Endpoints variables

Regarding the two main outcomes considered, change in SBP and total TC from baseline to follow-up, we extracted the mean difference and standard deviation of mean difference in the intervention and comparison group. As for change in smoking habits, we registered the number and frequency of smokers at baseline and follow up in each trial arm. Investigators were contacted for additional data when necessary.

A secondary outcome was change in estimated total cardiovascular risk from applied algorithms (i.e. Framingham, PROCAM). In interpreting the available data disagreements were resolved by discussion among the three reviewers (HMB, TOK, IS) and subsequent consensus.

### Risk of bias in individual studies

Three reviewers (HMB, TOK, IS) independently assessed potential sources of bias specific to RCTs using the Cochrane Collaboration’s tool [15]. Trials were classified as having an overall low risk of bias when the following core domains were judged at low risk of bias; concealment of randomization, blinding of outcome assessor, and intention-to-treat analyses.

### Quantitative data synthesis

#### Statistical pooling

In order to calculate an overall effect of intervention, the total standardized mean difference (SMD) with 95% confidence interval (CI) was estimated (Cohen´s method). If the value of zero is not included in the 95% CI, then the SMD is statistically significant at the 5% level (*p* < 0.05). Recommended interpretation is that a value of 0.2 indicates a small effect, 0.5 a medium effect, and of ≥ 0.8 a large effect. Fixed and random effects model analyses were considered, and in presence of heterogeneity between trials we used the DerSimonian and Laird method [16].

#### Sources of heterogeneity, evaluation and quantification

Statistical heterogeneity among studies was assessed with Cochran’s Q test. The magnitude of heterogeneity was evaluated by the I^2^ statistics which describes the proportion of total variation due to heterogeneity rather than chance [17]. Potential sources of heterogeneity were investigated first by subgroup analyses. We stratified our data according to type of intervention (physical activity, diet, and both), and the following study characteristics: concealment of randomization, blinding of the endpoint assessors on allocated treatment and analysis according to intention-to-treat strategy. We extended the analyses by a random-effect meta-regression where the outcome variable was the observed SMD from every study, indicating treatment effect, and the different patient- and study-level characteristics (covariates). Source of heterogeneity was considered as important if the covariate decreased the between-study variance. The estimate of τ^2^, in the presence of a covariate in comparison to that when the covariate is omitted, allows the proportion of the heterogeneity variance explained by the covariate to be calculated [18].

A sensitivity analysis was undertaken to investigate the influence of each study by omitting each in turn from the meta-analysis, and assessing the degree to which the magnitude and significance of the intervention effect changed [19].

#### Small-study effect

Small-study effect was evaluated visually by the funnel-plot and by Egger’s test of asymmetry applied on the funnel-plot [20].

All statistical analyses were performed with STATA 15.0 [21] and R Package-meta [22].

## Results

### Study selection

**Fig. 1:**
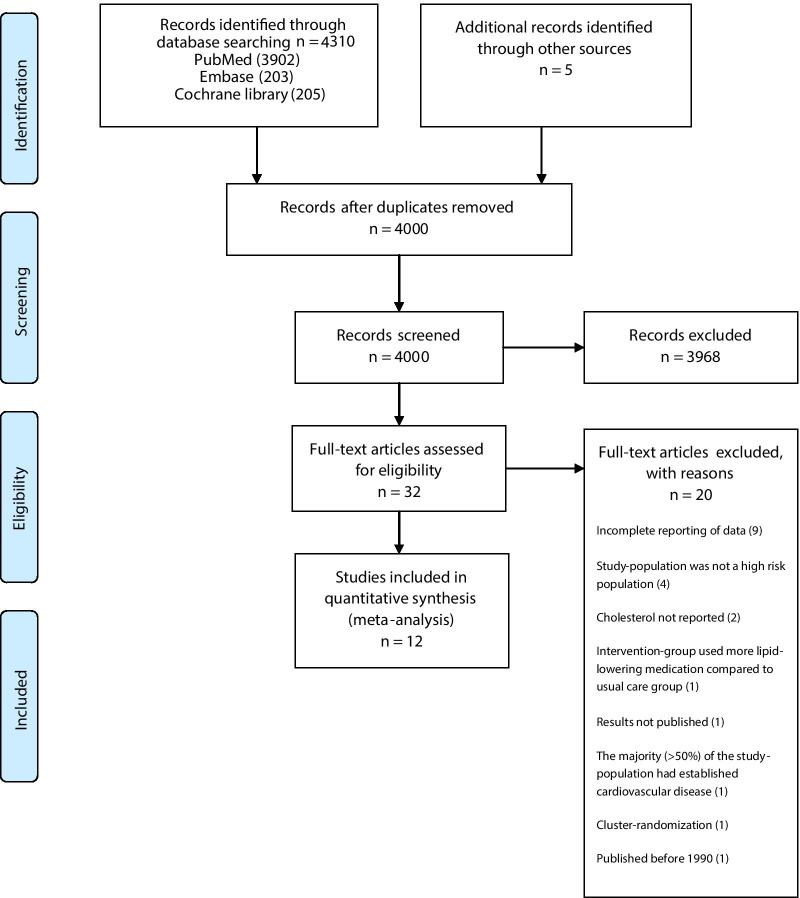
PRISMA flow diagram of study selection from the literature searches for the systematic review of randomized clinical trials investigating the efficacy of long-term lifestyle intervention on major cardiovascular risk factors in adult high-risk subjects

After identifying 4315 references, 4283 were excluded due to irrelevant content and duplicate publication, leaving 32 potentially eligible studies that were examined more thoroughly. After evaluating the full texts of these 32 publications, twenty articles were excluded and twelve studies [23–34] were finally included in our meta-analysis (Fig. 1).

For six trials we obtained additional endpoint measures by communication with an author of these studies [23, 24, 26, 27, 29, 34]. We estimated the standard deviation of mean difference from the reported standard error for two trials [31, 32] and from the 95% confidence interval for one trial [33]. For one trial we did not obtain a clarification from the authors whether the measure of variability presented was the standard deviation or the standard error [25]. We assumed it was the standard error since their measure was ten times lower than the standard deviation of the eleven other studies included in our meta-analysis.

### Study characteristics

The 12 studies included 6350 patients from three geographical regions; North-America (6 trials), Western-Europe (5 trials) and Oceania (1 trial). Patient-related characteristics at baseline are shown in Table 1. The median age of the patients was 54 years (range 44–70), 37.9% were males (range 0–100) and 11.3% smokers (range 0–34). The median baseline value for SBP was 129.8 mmHg (range 119.3–149), TC 5.5 mmol/l (range 4.4–6.3) and BMI 30.3 kg/m^2^ (range 26.7–38.8). Median follow-up time was 24 months (range 24–36). Details concerning intervention components and study populations are given in Table 2.

**Table 1:** Baseline characteristics of the 12 studies included in the meta-analysis

First author (references)	Intervention/comparison (n/n)	Follow-up (months)	Age (years) Mean	Males (%)	BMI Mean	Smokers (%)	SBP Mean	TC Mean
Simons-Morton [34]	289/292	24	51.3	54.7	29.8	8.4	119.3	–
Lindström [30]	265/257	24	55.0	33.0	31.2	–	138.0	5.60
Esposito [25]	90/90	24	43.9	55.0	28.0	0	135.0	5.08
Hjerkinn [27]	139/142	36	70.0	100	26.7	34.0	149.0	6.30
Ratner [31]	1079/1082	24	50.4	31.5	34.1	6.7	123.5	5.3
Anderssen [23]	142/143	24	57.0	100	29.2	15.8	140.3	5.97
Lawton [29]	544/545	24	58.9	0	29.2	12.6	123.1	6.07
Eriksson [24]	71/74	24	54.4	42.8	29.8	20.7	145.5	5.46
Kuller [28]	253/255	30	57.0	0	30.8	6.0	124.1	5.51
Vetter [32]	131/130	24	51.9	20.3	38.8	10.0	121.9	4.85
Goyer [26]	62/62	24	54.4	67.8	31.9	21.8	137.5	5.45
Lee [33]	103/104	24	51.7	20.3	36.6	–	124.5	4.4

BMI, body mass index (kg/m^2^); SBP, systolic blood pressure (mmHg); TC, total cholesterol (mmol/l)

**Table 2:** Study characteristics

Study	Year	Country	Population	Intervention	Comparison
Simons-Morton [34]	2001	USA	Inactive adults	Physical activity Advice/counseling, telephone contacts, interactive mail-contacts, self-monitoring devices	Physician advice
Lindström (30)	2003	Finland	Overweight/obese adults with IGT	Physical activity and diet. Advice/counseling, exercise sessions	General information
Esposito [25]	2004	Italy	Sedentary adults with metabolic syndrome	Diet Advice/counseling	General information + bi-monthly sessions
Hjerkinn [27]	2005	Norway	Elderly men with long standing hyperlipidemia	Diet Advice/counseling, placebo capsules (omgega-3)	Placebo-capsules (omega-3)
Ratner [31]	2005	USA	Overweight adults with IGT	Physical activity and diet Advice/counseling, exercise sessions	General information + annual individual sessions + placebo (Metformin)
Anderssen [23]	2005	Norway	Sedentary, overweight/obese men on antihypertensive drug treatment, elevated TC	Physical activity and diet Advice/counseling, exercise sessions, placebo (Fluvastatin)	Usual care by own general practitioner + placebo (Fluvastatin)
Lawton [29]	2008	New Zealand	Inactive women	Physical activity Advice/counseling, telephone contacts	Usual care from primary care practice
Eriksson [24]	2009	Sweden	Adults with hypertension, dyslipidemia, T2DM, obesity or any combination thereof	Physical activity and diet Advice/counseling, exercise sessions	General information + examination at 3, 12, 24 and 36 months
Kuller [28]	2012	USA	Overweight/obese women	Physical activity and diet Advice/counseling	6 Seminars first year with focus on women`s health, several times per year thereafter
Vetter [32]	2013	USA	Obese adults with at least one other criterion for metabolic syndrome	Physical activity and diet Advice/counseling	Quarterly primary care provider visits
Goyer [26]	2013	Canada	Adults with BMI ≥ 30 kg/m^2^ and at least 2 cardio-vascular risk factors: elevated SBP or DBP, elevated LDL, elevated TC/HDL, uncontrolled T2DM, smoking	Physical activity, diet and stress management Advice/counseling, exercise sessions	Usual care (family physician). At 1 year contact for address verification + reminder of 2 year follow-up
Lee (33)	2015	USA	American Indians with BMI ≥ 25 and metabolic syndrome	Physical activity and diet Advice/counseling, exercise sessions	Information by mail. Follow-up assessments every 6 months

IGT, impaired glucose tolerance; TC, total cholesterol; T2DM, type 2 diabetes mellitus; BMI, body mass index; SBP, systolic blood pressure; DBP, diastolic blood pressure;

### Risk of bias

As shown in Table 3 randomization and adequate concealment were present in six trials [24–26, 28, 29, 34]. Blinding of participants and health professionals to intervention allocation throughout the twelve trials was impossible, due to the nature of the trials, and classified open labeled with potential risk of bias. Blinding of the endpoint assessor to treatment allocation was considered adequate in all trials for endpoint TC, as laboratory staff presumably was unaware of subject group assignment. For endpoint SBP, blinding was reported in two trials [29, 34]. Drop-out was present in eleven trials, ranging from 3 to 27% (median 11%). Intention-to-treat strategy was reported in ten trials [23–25, 28–34]. A priori power estimation was presented in nine trials, but only one was adequate for the endpoints considered in our meta-analysis [29]. In summary, two of the included trials represented high-quality according to concealment of randomization, blinding of outcome assessor and intention-to-treat analyses [29, 34].

**Table 3:** Authors’ risk of bias assessment for the 12 studies included in the meta-analysis

First author (references)	Selection bias		Performance bias	Detection bias		Attrition bias	Other sources of bias
	Random sequence generation	Allocation concealment	Blinding of participants and personnel^a^	Blinding of outcome assessment		Incomplete outcome data	Intention to treat analysis
				SBP	TC		
Simons-Morton [34]	Low	Low	High	Low	Low	Unclear	Low
Lindström [30]	Low	Unclear	High	Unclear	Low	Unclear	Low
Esposito [25]	Low	Low	High	Unclear	Low	Unclear	Low
Hjerkinn [27]	Unclear	Unclear	High	Unclear	Low	Unclear	Unclear
Ratner [31]	Unclear	Unclear	High	Unclear	Low	Unclear	Low
Anderssen [23]	Unclear	Unclear	High	Unclear	Low	Unclear	Low
Lawton [29]	Low	Low	High	Low	Low	Unclear	Low
Eriksson [24]	Low	Low	High	Unclear	Low	Unclear	Low
Kuller [28]	Low	Low	High	Unclear	Low	Unclear	Low
Vetter [32]	Low	Unclear	High	Unclear	Low	Unclear	Low
Goyer [26]	Low	Low	High	Unclear	Low	Unclear	Unclear
Lee [33]	Unclear	Unclear	High	Unclear	Low	Unclear	Low

Risk of bias graded as low, high, unclear

SBP, systolic blood pressure; TC, total cholesterol

^a^All trials were judged open labeled

### Synthesis of results

#### Endpoint SBP

**Fig. 2:**
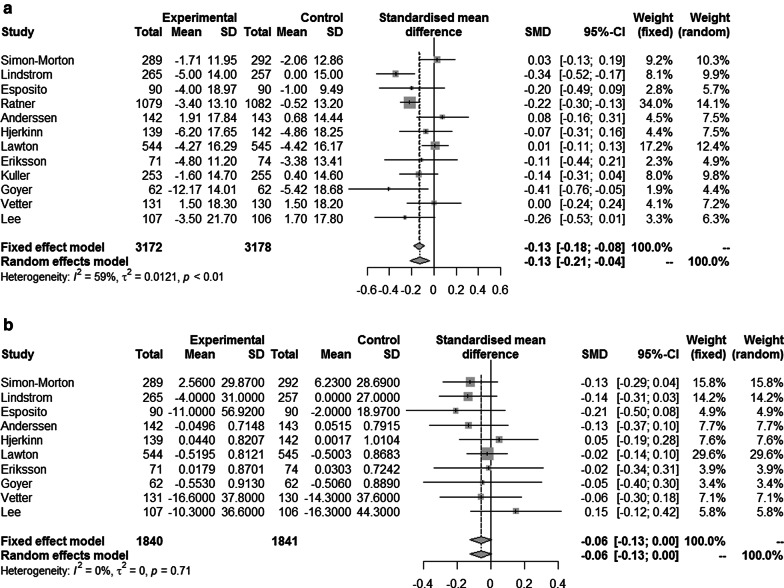
Forest plot for meta-analysis of randomized clinical trials comparing lifestyle interventions with usual care in adult high-risk subjects for endpoint; **a** systolic blood pressure, **b** total cholesterol

The pooled estimate from 12 studies indicated a small effect of lifestyle intervention on SBP (SMD = − 0.13, 95% CI − 0.21 to − 0.04, *p* = 0.0048), with moderate heterogeneity (I^2^ = 59%) (Fig. 2a). The absolute difference between the mean value in intervention versus comparison group was approximately 2 mmHg (MD = − 1.86, 95% CI − 3.14 to − 0.57, *p* = 0.0046).

**Fig. 3:**
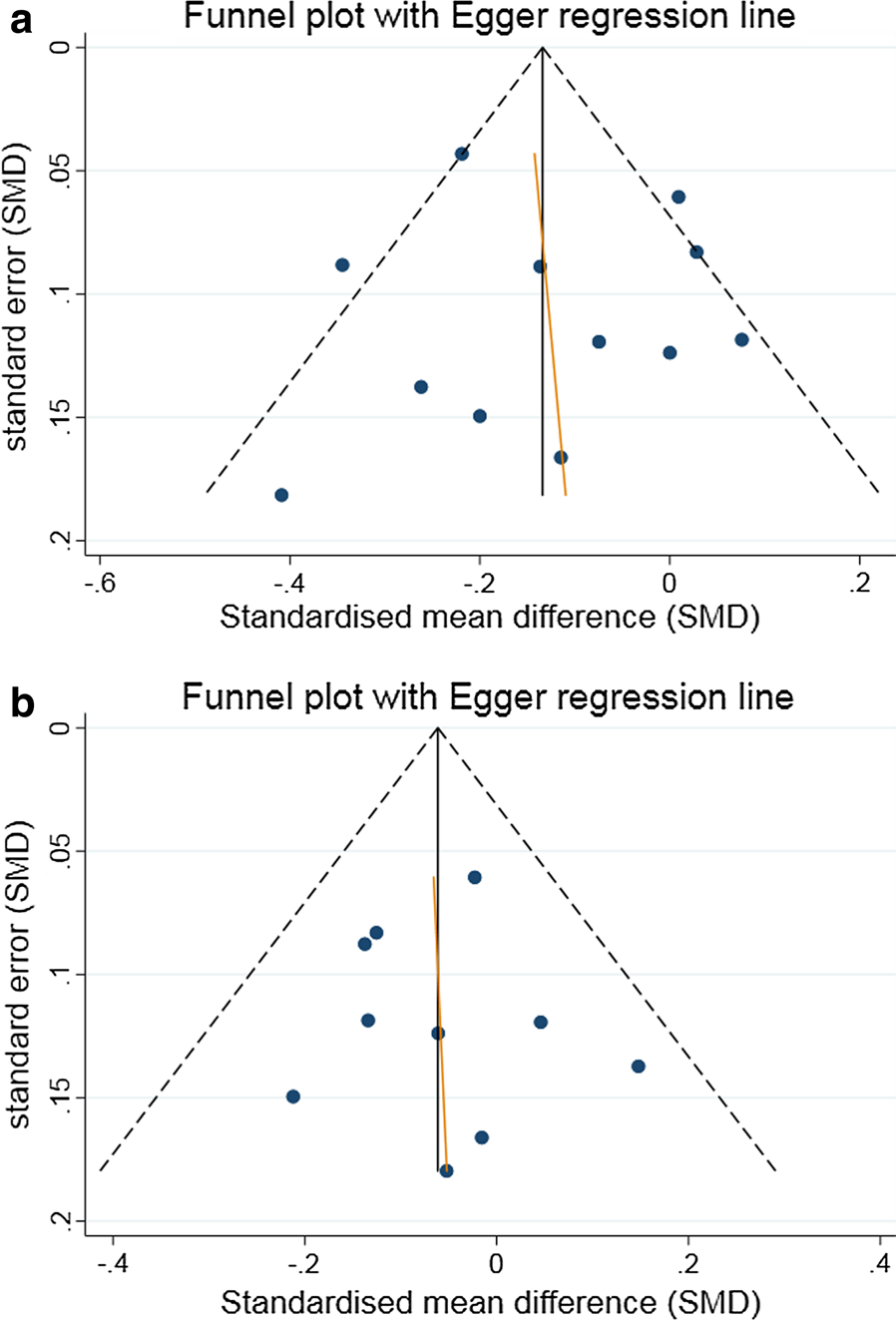
Funnel plot of the effect of lifestyle interventions for endpoint **a** systolic blood pressure, **b** total cholesterol

When stratifying on type of intervention, difference in the pooled estimates was mostly observed between the two groups consisting of physical activity only (SMD = 0.02, 95% CI − 0.08 to 0.11) or diet only (SMD = − 0.12, 95% CI − 0.31 to 0.06), and the group with a combination of physical activity and diet (SMD = − 0.18, 95% CI − 0.28 to − 0.08). Considering the trials satisfying three major parameters of internal validity, no effect of lifestyle intervention was demonstrated (SMD = 0.02, 95% CI − 0.08 to 0.11), while for the trials with non-presence of all three conditions a small effect was found (SMD = − 0.17, 95% CI − 0.25 to − 0.09). Extending the analysis with meta-regression, the study quality was associated with the effect of lifestyle intervention (*p* = 0.0136), accounting for 68% of the observed heterogeneity. None of the patient-related variables considered (mean age, mean BMI, frequency of male sex and frequency of smokers) were significantly associated with intervention effect. There was no indication of small-study effect as the funnel plot visually appeared symmetrical (Fig. 3a), supported by Egger’s test (*p* = 0.921). The robustness of the pooled effect was demonstrated by influential analysis. Whatever study we omitted, the pooled estimate did not change in magnitude, direction or statistical significance.

#### Endpoint TC

The pooled estimate from ten studies (Fig. 2b) indicated no significant effect of lifestyle intervention on total cholesterol (SMD = − 0.06 (95% CI − 0.13 to 0.00, *p* = 0.0634), with no heterogeneity (I^2^ = 0%). The absolute difference between the mean value in intervention versus comparison group was 0.05 mmol/l (MD = − 0.05, 95% CI − 0.11 to 0.00, *p* = 0.0495). Subgroup analysis and meta-regression were not indicated since there was no observed heterogeneity between the trials. No indication of small-study effect was found as the funnel-plot visually appeared symmetrical (Fig. 3b), confirmed by Egger’s test (*p* = 0.893). A stable pooled estimate was demonstrated by influential analysis, omitting one study at a time from the meta-analysis.

#### Smoking habits

After receiving follow-up information, four studies reported on change in smoking habits [24, 26, 31, 34]. Two of these studies [24, 26] demonstrated that the proportion of current smokers at follow-up were lower than at baseline in the intervention groups, while no change in the control groups.

#### Total cardiovascular risk

Only one study [26] reported on change in total cardiovascular risk applied from algorithms (Framingham, PROCAM). This trial showed that the 10-year Framingham cardiovascular risk declined from 16 to 14.6% in the intervention group compared to an increase from 14.3 to 19.1% in the control group, bearing in mind that the changes in both groups also were affected by the fact that the participants gained 2 years of age during the study. The PROCAM cardiovascular risk calculations declined in both groups.

## Discussion

The main result of this meta-analysis of RCTs examining long-term effects of lifestyle intervention indicates a limited effect on major cardiovascular risk factors SBP and TC. Regarding smoking cessation there was numerically greater reduction in smoking rates in the intervention groups compared to the comparison groups.

The limited long-term effect of lifestyle intervention may seem in contrast to the great importance lifestyle intervention has been given in guidelines on cardiovascular prevention [6–10]. As an example, the 2019 ACC/AHA Guideline reports that lifestyle intervention with 6 different elements including diet and exercise may each reduce SBP by at least 4 mmHg in hypertensive subjects and 2 mmHg in normotensives, suggesting larger effects of combined measures [9]. However, the guideline does not specify the duration of the expected effects. From the Look Ahead study [35], the challenge of maintaining the benefits of lifestyle intervention on blood pressure (and other parameters) is evident: At 12 months, SBP was reduced by 6,8 mmHg in the intensive lifestyle group compared to 2,8 mmHg in the standard care group. However, averaged across the first 4 years, the difference in SBP was reduced by 5.33 versus 2.97 mm Hg; i.e. a difference of 2.36 mmHg, very similar to the results in our meta-analysis.

A small effect on SBP, but no effect on TC, was similarly reported in the Finnish Diabetes Prevention Study [30], where SBP were lowered by 5 mmHg after 2 years while TC remained unchanged. Also the American Diabetes Prevention Program demonstrated significantly greater decrease in SBP in the lifestyle group (− 3.4 mmHg ± 0.4) compared to control group (− 0.52 mmHg ± 0.4), but no changes in the TC levels [31].

In the meta-analysis from 2018 by Sisti et al. [13], both SBP and TC were significantly lowered after 6 months. The decrease in SBP was − 5.20 mmHg (95% CI − 9.4, − 1.07) after 6 months and − 3.20 mmHg (95% CI − 4.56 to 1.84) after 12 months. TC was lowered by − 0.36 mmol/l (95% CI − 0.63 to 0.10) after 6 months and − 0.23 mmol/l (95% CI − 0.39 to 0.08) after 12 months. Almost identical findings were reported from Ebrahim’s Cochrane review [11] where the reduction of SBP was − 2.71 mmHg (95% CI − 3.49 to 1.93) while TC levels dropped by − 0.07 mmol/L (95% CI − 0.08 to 0.06) after 12 months.

In summary, major lifestyle studies and meta-analyses show that lifestyle intervention result in small, but significant changes in SBP and TC after 6–12 months. However, the benefits gradually attenuate over time, especially regarding TC. Our study confirms this attenuation of benefits and demonstrates further reductions in the period from 12 to 24 months.

Trials examining lifestyle intervention should ideally have clinical endpoints, as the ultimate goal is reducing cardiovascular events. Trials with clinical outcomes are large and costly, however, which explains why most lifestyle intervention trials instead focus on improvements in established cardiovascular risk factors. Reviews so far have reported effects after 3–12 months, and occasionally up to 18 months, motivating our attention to study the effects after longer follow-up. Ideally, however, even longer follow-up, i.e. 5–10 years, would be optimal to substantiate the clinical value of the interventions. From our literature search, such data appear very limited.

The present study demonstrates that a small reduction in SBP of approximately 2 mmHg may be maintained over time with lifestyle intervention. This was observed in a population with mean baseline SBP of 129 mmHg and many studies report that greater reductions may be achieved when the baseline blood pressure is higher [9, 36]. Although the effect of lifestyle intervention appears small, a reduction of this size may result in a valuable reduce in the risk of future CVD, according to epidemiological evidence and studies evaluating benefits of sodium reduction in the population [36], that suggested that a reduction of 3.8 mmHg could prevent 1,6 million annual CVD deaths globally.

While a limited effect on SBP was observed, we found no effect on cholesterol levels. The reasons for the lack of efficacy are unclear, but may relate to the intensity of the intervention, the quality of the dietary and exercise advice and to difficulties in maintaining lifestyle changes over time. Since several studies of short duration, i.e. 3–6 months, have reported considerably larger effects on blood pressure [37], and to some extent on cholesterol [38, 39], the latter explanation may appear the most important.

Traditional lifestyle interventions have had cholesterol levels and smoking cessation as the main targets [40, 41]. However, both smoking rates and cholesterol levels have been significantly reduced in most western populations the last decades, the latter mainly due to general improvements in the population diet, for example with reductions in trans fat [42]. Accordingly, reduction in cholesterol levels through diet modifications may have become harder to achieve. Hence, the most feasible target for risk reduction through lifestyle interventions in non-smokers may now have become a reduction in SBP.

The results in our meta-analysis highlight the need to develop strategies that enable high-risk individuals to maintain the effects on risk factors beyond 3–6 months. At present, we do not know if the attenuation of the effects is entirely related to a reduced compliance/patient fatigue regarding lifestyle habits, or if it may also represent some sort of physiological adaptation to the obtained lifestyle changes. A further possibility is that the advices used in the included studies are no longer optimal, and need to be revised according to the contemporary risk profile and lifestyle challenges in the population.

### Strengths and limitations

This meta-analysis is, to the best of our knowledge, the first addressing the impact of multiple lifestyle intervention on cardiovascular risk factors in the long-term; i.e. after 24 months.

Our review was based on a comprehensive literature search, which reduces the possibility of missing relevant trials [43]. Trial selection was done by two authors, and data extraction by three authors, to minimize transcription errors. As recommended by the Cochrane Collaboration tools for assessing risk of bias in randomized trials [15], we did not use summary scores to identify quality of trials. The components used for quality assessment are validated and reported to be associated with bias [44]. The analysis applied the recommended principles for meta-analysis methodology regarding eligibility criteria for the individual trials, analysis methods to explore sources of heterogeneity between studies and evaluate small-study effect [45].

Small-study effect was unlikely to affect our results. A major limitation was the observed heterogeneity between trials. Our results concerning the efficacy of lifestyle intervention on SBP was altered when stratifying on the components of trial quality, and meta-regression demonstrated that trial quality was an important determinant of the intervention effect. The impact of study-level variables on meta-analysis results has been investigated, indicating true associations between heterogeneous treatment effects and the study-level variables [46]. On the other hand, the diversity could be related to differences in the patient population studied and differences in interventions. The population in the two trials classified as having an overall low risk of bias [29, 34] was normotensive, making it less likely to expect a significant SBP reduction. Moreover, the intervention in these two trials consisted of physical activity only, which quite likely may have reduced the impact of the intervention compared to trials also including dietary advice.

The review considered trials published after 1990 only. Of the 4315 records identified, only 12 trials could be included, illustrating the sparse number of RCTs with follow-up time as long as 24 months and sufficient data to be evaluated.

### Future directions

A final answer on the efficacy of lifestyle interventions for reducing cardiovascular risk would require RCTs large enough to evaluate effects on clinical outcomes, but such trials would have to be very large and costly, as exemplified by the Look AHEAD study [35]. A more feasible approach could be the use of proper validated risk algorithms as primary outcomes, as these integrate the effects on multiple risk factors and allow valuable estimates of the intervention on total cardiovascular risk. Meanwhile, further trials should focus on the challenge of maintaining the benefits often reported in studies of 6–12 months duration. In this respect it is worth noting that the most feasible targets for risk reduction may now have become reduction in SBP and smoking cessation, as reductions in cholesterol levels through diet counseling seem hard to achieve.

## Conclusion

In conclusion, our results suggest that the effects of lifestyle intervention on major cardiovascular risk factors after 24 months of follow-up are limited, but a modest effect on SBP may be of clinical relevance. Our observations demonstrate the challenge of maintaining benefits during longer follow-up, and suggest a need to develop new strategies to promote durable changes in cardiovascular risk.

## Supplementary Information

**Additional file 1.** Title page, not relevant as supporting document as its content is covered in the title-page in the publication.

**Additional file 2.** Datafile on endpoint SBP.

**Additional file 3.** Datafile on endpoint SBP.

**Additional file 4.** Datafile on endpoint Tchol.

**Additional file 5.** Datafile on endpoint Tchol.

## Data Availability

All data generated and analysed during this study are included in this article and its supplementary information files.

## Abbreviations

CVD: Cardiovascular disease
TC: Total cholesterol
SBP: Systolic blood pressure
RCT: Randomized controlled clinical trial
BMI: Body mass index
SMD: Standardized mean difference
IGT: Impaired glucose tolerance
T2DM: Type 2 diabetes mellitus
DBP: Diastolic blood pressure
