# Supplementary material for: Long-term effects (> 24 months) of multiple lifestyle intervention on major cardiovascular risk factors among high-risk subjects: a meta-analysis
Source: BMC Cardiovasc Disord. 2021 Apr 15;21:181. doi: 10.1186/s12872-021-01989-5 (PMC8048075; doi:10.1186/s12872-021-01989-5)
Supplement: Supplementary file 1 — Additional file 1. Title page, not relevant as supporting document as its content is covered in the title-page in the publication. [file 12872_2021_1989_MOESM1_ESM.docx]

**Long-term effects (>24 months) of multiple lifestyle intervention on major cardiovascular risk factors among high risk subjects: a meta-analysis**

Hilde Bergum, Department of rehabilitation and lifestyle medicine, LHL-Hospital Gardermoen, Norway

Irene Sandven, Oslo Centre for Biostatistics and Epidemiology (OCBE), Oslo University Hospital, Norway

Tor Ole Klemsdal, Department of Preventive Cardiology, Oslo University Hospital, Norway

**Corresponding author:**

Hilde Bergum, Department of rehabilitation and lifestyle medicine, LHL-Hospital Gardermoen, Postboks 103 Jessheimbyen, 2051 Jessheim, Norway

Email: hilde.bergum@lhl.no
